# A Comprehensive Gene Expression Meta-analysis Identifies Novel Immune Signatures in Rheumatoid Arthritis Patients

**DOI:** 10.3389/fimmu.2017.00074

**Published:** 2017-02-02

**Authors:** Sumbul Afroz, Jeevan Giddaluru, Sandeep Vishwakarma, Saima Naz, Aleem Ahmed Khan, Nooruddin Khan

**Affiliations:** ^1^Department of Biotechnology and Bioinformatics, School of Life Sciences, University of Hyderabad, Hyderabad, India; ^2^Centre for Liver Research and Diagnostics, Central Laboratory for Stem Cell Research and Translational Medicine, Deccan College of Medical Sciences, Kanchanbagh, Hyderabad, India

**Keywords:** rheumatoid arthritis, microarrays, meta-analysis, synovial inflammation, autoimmunity

## Abstract

Rheumatoid arthritis (RA), a symmetric polyarticular arthritis, has long been feared as one of the most disabling forms of arthritis. Identification of gene signatures associated with RA onset and progression would lead toward development of novel diagnostics and therapeutic interventions. This study was undertaken to identify unique gene signatures of RA patients through large-scale meta-profiling of a diverse collection of gene expression data sets. We carried out a meta-analysis of 8 publicly available RA patients’ (107 RA patients and 76 healthy controls) gene expression data sets and further validated a few meta-signatures in RA patients through quantitative real-time PCR (RT-qPCR). We identified a robust meta-profile comprising 33 differentially expressed genes, which were consistently and significantly expressed across all the data sets. Our meta-analysis unearthed upregulation of a few novel gene signatures including *PLCG2, HLA-DOB, HLA-F, EIF4E2*, and *CYFIP2*, which were validated in peripheral blood mononuclear cell samples of RA patients. Further, functional and pathway enrichment analysis reveals perturbation of several meta-genes involved in signaling pathways pertaining to inflammation, antigen presentation, hypoxia, and apoptosis during RA. Additionally, PLCG2 (phospholipase Cγ2) popped out as a novel meta-gene involved in most of the pathways relevant to RA including inflammasome activation, platelet aggregation, and activation, thereby suggesting PLCG2 as a potential therapeutic target for controlling excessive inflammation during RA. In conclusion, these findings highlight the utility of meta-analysis approach in identifying novel gene signatures that might provide mechanistic insights into disease onset, progression and possibly lead toward the development of better diagnostic and therapeutic interventions against RA.

## Introduction

Rheumatoid arthritis (RA) is a chronic, progressive, and inflammatory autoimmune disease, which continues to cause global disability having a worldwide prevalence of 1% (1). The disease is classified primarily by clinical phenotype and predominantly associated with articular, extra-articular, and systemic complications including anemia, cardiovascular diseases, osteoporosis, fatigue, and depression (2, 3). There exists a considerable geographical and temporal variability as well as unpredictability based on severity and manifestations in the occurrence of RA within populations (4). RA is characterized by synovial inflammation [excessive infiltration and activation of neutrophils, mononuclear cells including T cells, B cells, plasma cells, and mast cells (5), as well as complement factors such as C5a at rheumatoid joints (6)], autoantibody generation (rheumatoid factor and anti-citrullinated protein antibody) (3) and degradation of bones and cartilage leading to bone deformity (5). The etiology or cause of RA remains elusive. However, various studies have illustrated a convoluted interplay between genetic factors and environmental exposures in most of the disease cases (3, 7). Unprecedented efforts have been made to identify multitude of factors that have foremost allusions in RA pathogenesis including pro-inflammatory cytokines (TNF-α, IL-6, IL-17, and IL-1) (8) as the orchestrator of vital processes during RA progression involving synovitis (9), angiogenesis (production of pro- angiogenic factors particularly VEGF) (10, 11), osteoclastogenesis (increased production of macrophage colony-stimulating factor and RANK ligand) (12), cartilage degradation (enhanced secretion of MMPs into synovial fluid at the joints) (9, 13), and acute-phase proteins production such as CRP (exacerbates RA-related tissue damage) (14).

On the genetic front, the strongest link in RA has been ascribed to the third hypervariable region of the HLA-DRβ chains (aa. 70–74) within MHC. The active epitope within the area, glutamine–leucine–arginine–alanine–alanine (QKRAA) or QRRAA has been found to be associated with multiple RA allied DR genes including DR1, DR4, and DR14 (15). The location of the epitope within HLA-DR determines the specificity of the peptides presented to CD4^+^ T-cells. However, the specific peptides that bind to DR proteins in RA patients have not yet been identified (6). The abundance of effector T cells and IL-17-producing Th17 cells in the synovial milieu emphasize the critical role of these cells in RA pathogenesis. The activation of T-cells through HLA-DR4 (present on APCs) mediated presentation of specific peptides during RA initiates an array of complex immune responses including T-cell oligoclonality, germinal center reactions, and B-cell hypermutation highlighting local antigen-specific T-cell-mediated B-cell responses (3). The appropriate interconversion between Th1/2/17/Treg phenotypes has crucial implication toward RA outcome (16). In particular, an imbalance between Th17 and Treg cells has an impact on local TNF levels in the synovial fluid that blocks the differentiation of Treg cells and along with IL-17, macrophage, and dendritic cells derived TGF-β and IL-1β, shifts the T-cell homeostasis toward inflammation (17). The importance of B-cells in RA have been highlighted in autoantigen presentation and cytokine production (IL-6, TNF, and lymphotoxin-β) (3). Apart from acting as the precursor for autoantibody-generating plasma cells, B-cells are the only antigen-presenting cells supporting the activation of autoreactive T-cells during RA (18).

Although several factors have been identified, yet there is a dearth of knowledge about the complex gene networks associated with the disease. Over the last two decades, DNA microarray technology allowed us to interrogate thousands of genes simultaneously enrooting the discovery of disease-relevant genes. Multiple microarray experiments have been conducted to identify potential gene signatures that are responsible for the pathogenesis of various diseases. The analyses of these experiments usually generate hundreds of differentially expressed genes (DEGs), making it difficult to generate a convincing transcriptome profile of a particular disease condition. To address this issue, we implemented a meta-analysis method to extract a global transcriptional profile of RA patients available on public databases. Our gene expression meta-analysis revealed some credible gene signatures that have shown relevance to RA condition. Finally, we went a step further to validate these gene signatures in RA patient samples obtained clinically. In conclusion, this meta-analysis approach of publicly available microarray data suggests us to generate a significant gene expression profile that might represent an early step toward enhancement in the detection, treatment, and prevention of RA.

## Materials and Methods

### Data Sets Review

A comprehensive search was administered for RA gene expression data sets available on Gene Expression Omnibus (GEO) database. Independent of age, gender, race, and region, two sample groups, i.e., RA patients and healthy controls (HCs) were considered for this study. After a thorough search, we identified eight data sets, out of which two data sets (GSE25160, GSE42296) (19, 20) had a sample source of peripheral blood mononuclear cells (PBMCs) and six data sets (GSE1919, GSE12021, GSE48780, GSE55235, GSE55457, GSE55584) (21–24) had a sample source of synovial tissue. These eight data sets were subjected to independent differential gene expression analysis followed by a meta-analysis. The GEO accession IDs and samples information in each data set is summarized in Table 1.

**Table 1:** **GEO data sets and samples summary**.

Data set ID	GEO accession	Sample source	Rheumatoid arthritis samples	Healthy samples
1	GSE1919	Synovial tissue	5	5
2	GSE12021	Synovial tissue	13	24
3	GSE25160	Peripheral blood mononuclear cells (PBMCs)	13	4
4	GSE42296	PBMCs	19	4
5	GSE48780	Synovial tissue	27	6
6	GSE55235	Synovial tissue	10	10
7	GSE55457	Synovial tissue	10	13
8	GSE55584	Synovial tissue	10	10

### Data Sets Analyses

The raw data (.CEL files) of each data set was downloaded from GEO accession links. As the eight data sets were of Affymetrix platform, a single normalization method (RMA) was performed on the raw data. Samples of each data set were categorized into two groups; RA patients and HCs. The differential expression of genes between the two groups (RA vs. HC) was calculated using a moderated *T*-test, assigning a specific threshold (*p*-value < 0.05). To bypass the multiple testing problems, we adjusted the *p*-values using an optimized false discovery rate (25). Genes with *q*-values (adjusted *p*-values) less than 0.05 were considered as DEGs. The DEGs of each data set were annotated with Entrez IDs, official gene symbols, and gene names. All the analyses were performed in R using various Bioconductor packages (26, 27).

### Meta-analysis

Consequent to the individual data sets analyses, eight sets of DEGs were obtained and assigned to the meta-analysis study. The primary task of our meta-analysis method was to extract the intersected DEGs among the set of DEGs. The step by step automated meta-analysis method was as follows: (i) extract the intersected genes (meta-genes), (ii) exclude genes with inconsistent expression, and (iii) check for significance by combining adjusted *p*-values of each gene from all the data sets. The weighted *Z*-method was used to combine the individual *q*-values of each gene, where each test was assigned as weight (*w_i_*) and implemented using *survcomp*, an R package (28).

$$\text{Weighted}\;Z\text{-method},Zw=\frac{\sum_{i=1}^{k}w_{i}Z_{i}}{\sqrt{\sum_{i=1}^{k}w_{i}^{2}}}$$

The meta-analysis algorithm was implemented in R.

### GO Functional and Pathway Enrichment

The obtained 33 meta-genes were subjected to GO functional and pathway enrichment analysis. Biological processes and molecular functions of meta-genes were generated using Enrichr (http://amp.pharm.mssm.edu/Enrichr/), a web-based online tool (29). Pathway enrichment analysis of meta-genes was performed using ReactomeFIViz (30), a Cytoscape plug-in based on Reactome pathway database (31).

### Specimen Collection

Blood samples were collected from the individuals clinically diagnosed with RA reporting to Owaisi Hospital and Research Centre, Hyderabad, India. The procedure of the entire study was carried out by the protocols approved by the Institute Ethics Committee, University of Hyderabad. Written informed consent was taken from each patient, and the complete clinical information and medical history was well documented.

### PBMCs Isolation

For PBMCs isolation, blood samples were collected in the sterile EDTA Vacutainer blood collection tubes. PBMCs from the whole blood of HCs and RA patients were isolated using Ficoll Histopaque (Sigma Aldrich Company, UK) density gradient method as described earlier (32). Briefly, 1.5 ml of whole blood was diluted with 1× PBS-EDTA in a 1:1 ratio, was layered upon 1 ml Ficoll, and centrifuged at 400 × *g* for 30 min. The buffy layer was carefully removed and washed with 1× PBS-EDTA twice at 200 × *g* for the removal of blood platelets. Isolated PBMCs were suspended in TRIZOL (Invitrogen, Life Technologies) reagent for RNA extraction.

### Quantitative Real-Time PCR (RT-qPCR)

Total RNA from isolated PBMCs was extracted using TRIZOL reagent. RNA was reverse transcribed into cDNA using VERSO cDNA synthesis kit (Thermo Scientific) according to manufacturer’s instructions. As template, approximately 30 ng cDNA was used for RT-qPCR and the signals were detected using real-time PCR system. Quantitative real-time PCR was performed using Mastercycler ep realplex (Eppendorf). The cDNA was amplified using SYBR Green Mix (Kapa Biosystems) with gene-specific primers (Table S4 in Supplementary Material). The thermal cycler parameters followed are as follows: one cycle of 94°C for 2 min followed by 40 cycles of 30 s at 94°C, 30 s annealing at 56°C and 40 s at 68°C. The relative mRNA expression of each gene was calculated using housekeeping gene β-actin as the reference gene (33).

## Results

### Computational Analysis

#### Preliminary Analyses

**Figure 1:**
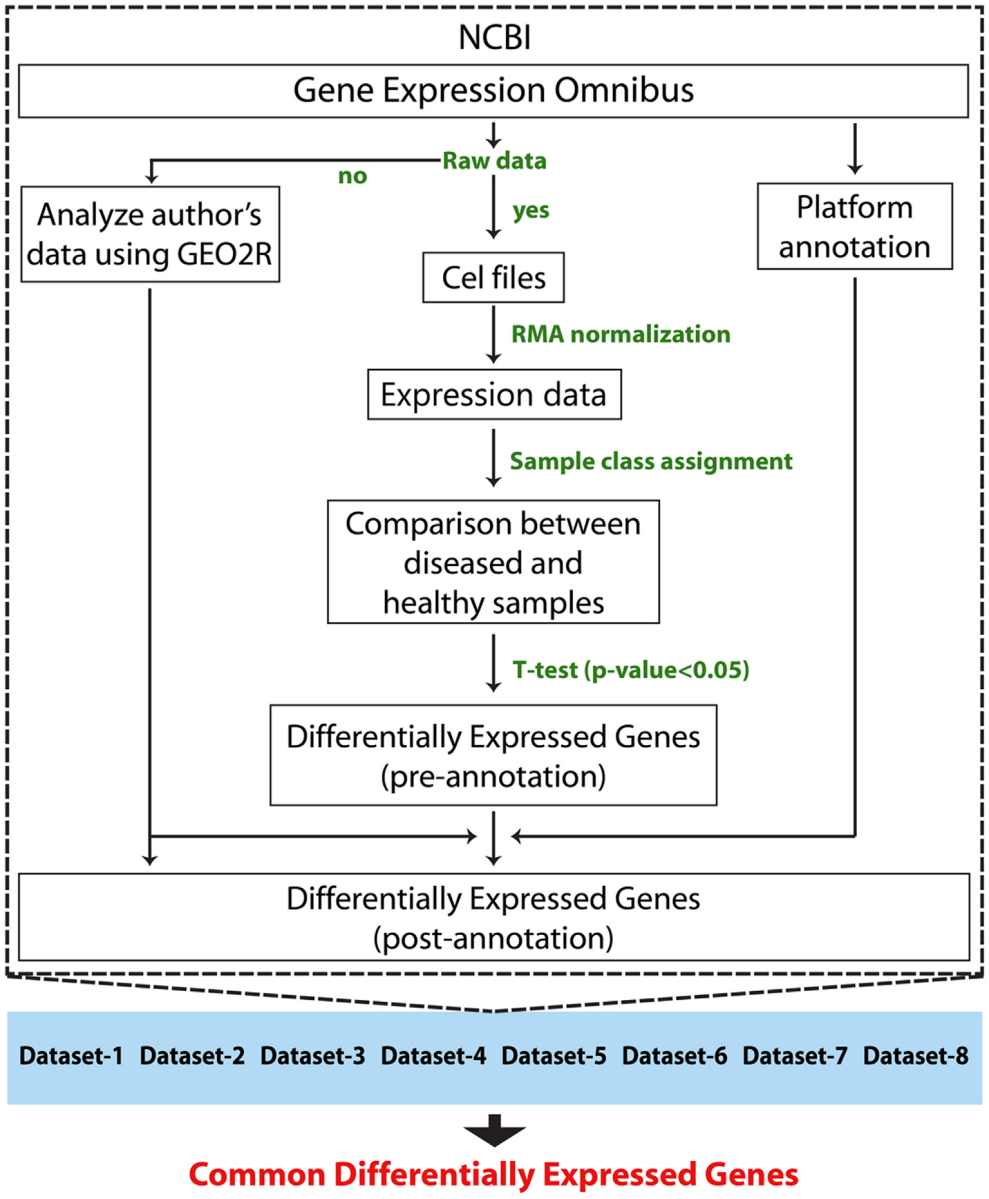
**Schematic representation of individual data set analysis followed by meta-analysis method**.

Followed by a complete systemic review of RA data sets available on GEO database, we downloaded raw data of eight data sets. Sample source of six data sets was of synovial tissue, and the remaining two data sets were of PBMCs (Table 1). In total, the study included 107 RA patients and 76 HCsamples. Before the meta-analysis study, we analyzed and generated the results of individual data sets to obtain eight set of DEGs. The raw data of each data set were normalized using RMA method. Later, the differential expression of each gene in each study was calculated for patients group with respect to the healthy group using a *T*-test statistic. Genes with *p*-values less than 0.05 were assigned as DEGs. Adjusted *p*-values (*q*-values) were calculated using Benjamini Hochberg’s method (25) to eliminate the false positives that arise due to multiple testing. The DEGs of each study were annotated with Entrez IDs, official gene symbols, and gene names. A total of 245,672 were measured, out of which 84,436 have shown a significant change in expression. We obtained eight sets of DEGs that were assigned for our meta-analysis study. The schematic representation of the analyses method is shown in Figure 1.

#### Meta-analysis

**Figure 2:**
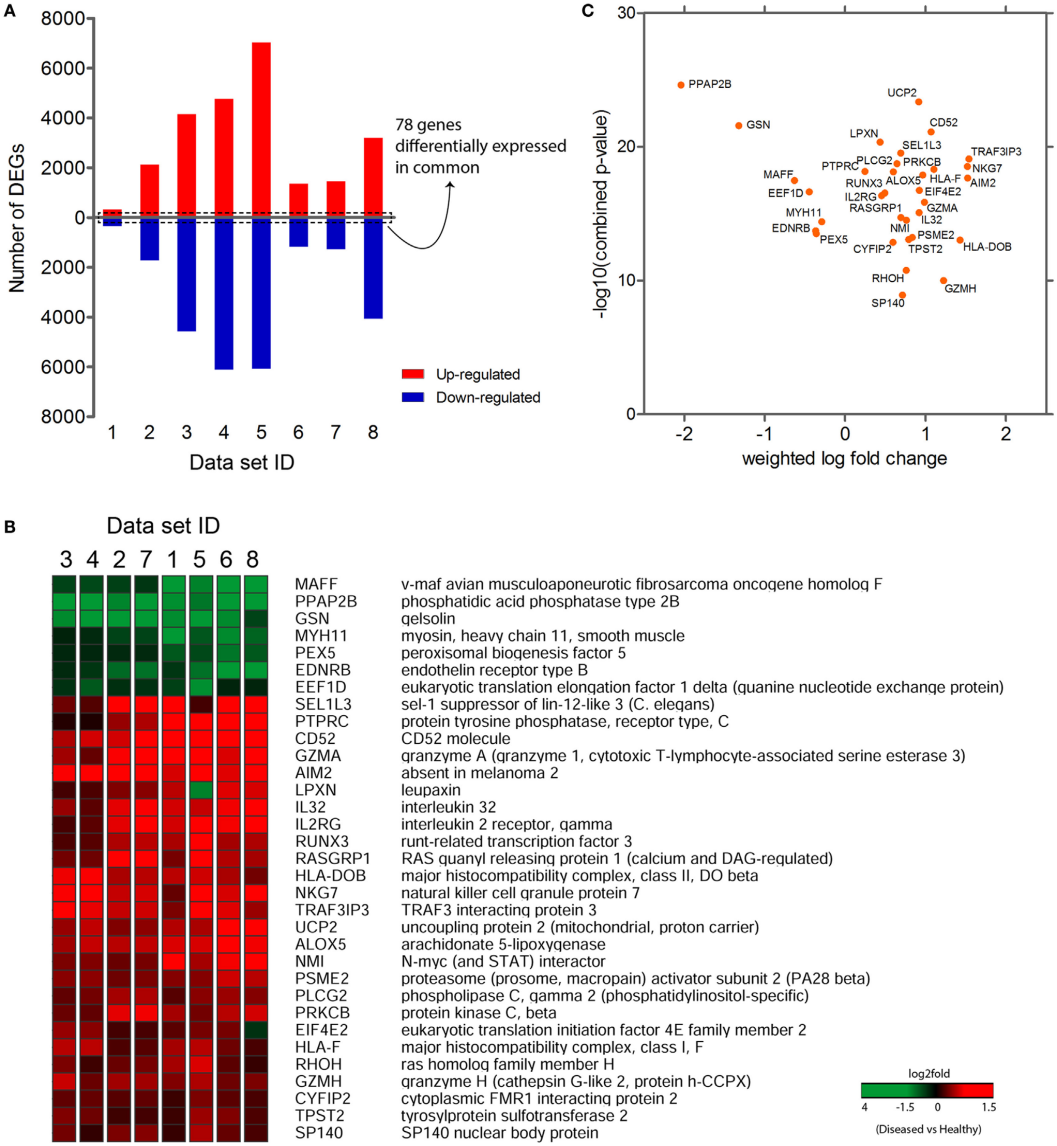
**Common differentially expressed genes (common DEGs)**. **(A)** 78 genes commonly expressed across eight data sets. **(B)** 33 consistently expressed DEGs (either completely upregulated or downregulated) at least in 7 data sets. **(C)** Graph plotted between the weighted log-fold change score and −log 10 (combined *p*-values).

The purpose of performing meta-analysis was to elicit the intersection of significant gene signatures (meta-profile) from the sets of DEGs obtained during our preliminary analyses. Despite the availability of various established microarray meta-analysis methods for the detection of common DEGs (meta-genes) (34), we sought to use a specific meta-analysis approach, where we combined the statistical parameters (*q*-values) obtained from differential gene expression analysis of individual data sets. After our preliminary analyses, we extracted DEGs that were commonly expressed across all data sets. We identified 78 genes that were commonly expressed in all eight data sets (Figure 2A), out of which 33 genes were consistently expressed (either upregulated or downregulated) in at least seven out of eight data sets (Figure 2B). In contrast, 34 out of remaining 45 genes have shown a consistent reverse expression between PBMCs and synovial tissue data sets (Figure S1 in Supplementary Material). This variation might be possible due to the difference in cellular tropism and secretion of various kinds of chemokines and cytokines in the synovium as compared to PBMCs. Also, the overall systemic responses occurring in blood against the localized responses generated in synovium might influence the expression level of a particular gene in both these areas. In the next step, we computed combined *p*-values of consistently expressed genes using weighted *Z*-method from the obtained independent *q*-values of each data set. The genes were ranked based on the combined *p*-values, thus evaluating the significance level of each gene. Also, a weighted log-fold change score was calculated for the list of genes and were plotted against the combined *p*-values (Figure 2C). In total, we observed 7 downregulated and 26 upregulated genes in the list of consistently expressed genes that were finally assigned as our candidate meta-signatures. As an adjunct analysis, a meta-analysis was performed for seven data sets (excluding the data set with least number of DEGs, i.e., data set 1) to obtain additional number of common DEGs (Figure S2 in Supplementary Material).

#### GO Enrichment Analysis

We performed a GO enrichment analysis to the 33 meta-signatures using Enrichr (29), an online tool based on Gene Ontology. Enrichment analysis was performed for two GO categories: biological process and molecular function. The top enriched GO terms for biological process were: regulation of B cell receptor (BCR) signaling pathway (GO: 0050855), cellular calcium ion homeostasis (GO: 0006874), mast cell activation (GO: 0045576), leukocyte activation (GO: 0045321), positive regulation of T cell-mediated cytotoxicity (GO: 0001916), cytolysis (GO: 0019835), immune response-regulating cell surface receptor signaling pathway (GO: 0002768), and regulation of protein oligomerization (GO: 0032459). The top enriched GO terms for molecular functions included peptide binding (GO: 0042277), amide binding (GO: 0033218), translation factor activity, nucleic acid binding (GO: 0008135), type 1 angiotensin receptor binding (GO: 0031702), angiotensin receptor binding (GO: 0031701), MHC class II receptor activity (GO: 0032395), and RNA cap binding (GO: 0000339). Detailed enrichment tables are provided in Tables S1 and S2 in Supplementary Material.

#### Pathway Enrichment Analysis

We performed pathway enrichment using ReactomeFiViz plug-in based on Reactome pathways database. A total of 20 out of 33 genes were involved in various pathways. The hit genes list comprised AIM2, ALOX5, CYFIP2, EDNRB, EEF1D, EIF4E2, GSN, HLA-DOB, HLA-F, IL2RG, MAFF, MYH11, PLCG2, PPAP2B, PRKCB, PSME2, PTPRC, RASGRP1, RHOH, and ST6GAL1. Various immune system pathways such as platelet activation, signaling and aggregation, the AIM2 inflammasome, downstream signaling events of BCR, signaling by the BCR, ER-phagosome pathway, and antigen processing cross presentation were enriched. The other distinct pathways include platelet activation, signaling and aggregation, GPVI-mediated activation cascade, hemostasis, synthesis of lipoxins (LX), synthesis of 5-eicosatetraenoic acids, disinhibition of SNARE formation, and signaling by VEGF. Top enriched pathway table is provided in Table S3 in Supplementary Material.

### Validation of Meta-analysis Data

#### RT-qPCR Analysis of Selected Genes

**Figure 3:**
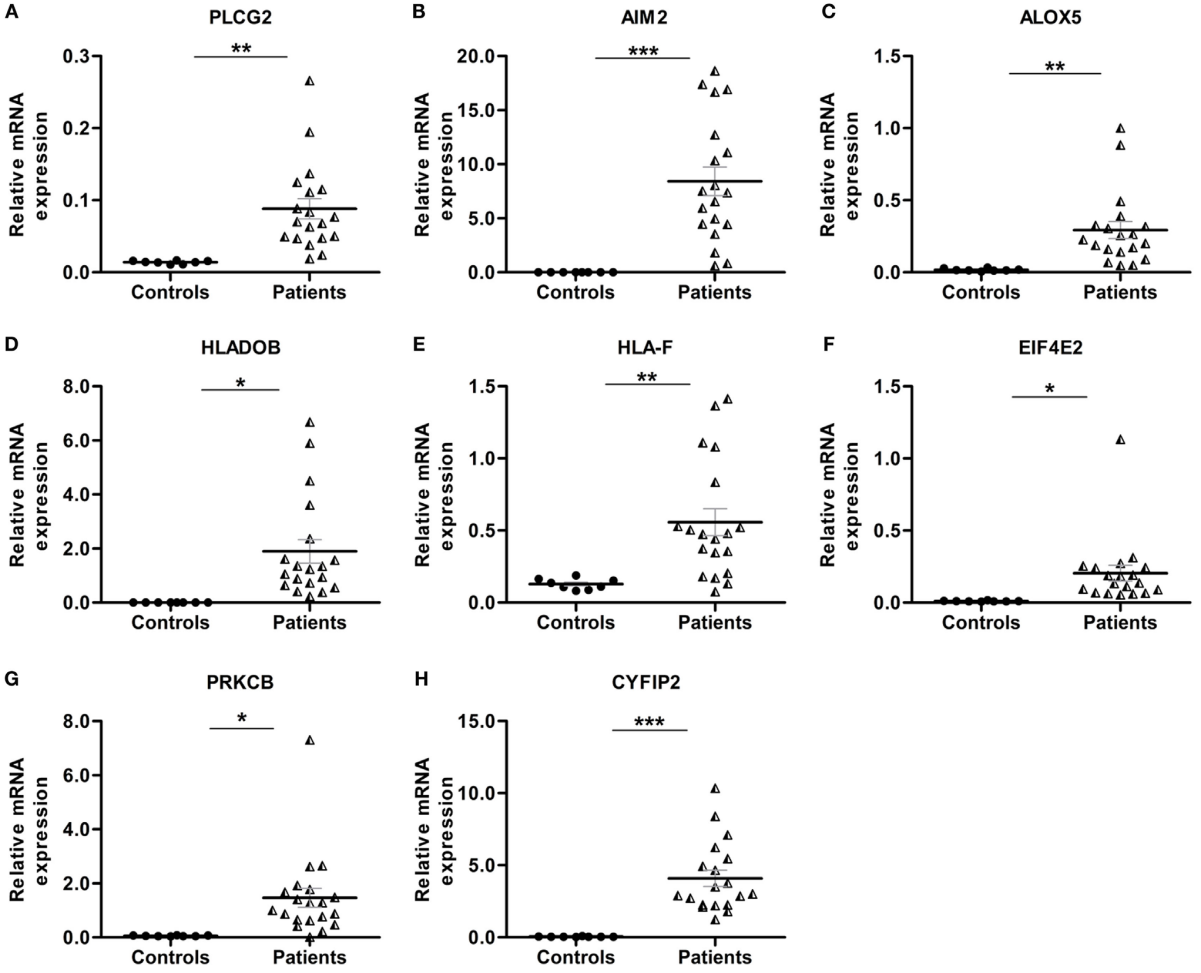
**Relative quantification of transcription of eight candidate genes (PLCG2, AIM2, ALOX-5, HLA-DOB, HLA-F, EIF4E2, PRKCB, and CYFIP2) in rheumatoid arthritis (RA) patient samples**. Quantitative real-time polymerase chain reaction (RT-qPCR) was carried out to quantify the relative expression of the above mentioned candidate genes in RA patients (*n* = 19) peripheral blood mononuclear cells compared to healthy controls (HCs) (*n* = 8). The relative expression of each gene **(A–H)** PLCG2, AIM2, ALOX-5, HLA-DOB, HLA-F, EIF4E2, PRKCB, and CYFIP2 was normalized to housekeeping gene β-actin. Experiments were carried out at least in triplicates. Error bars represent the SEM. *p* values were determined based on comparison with HCs. Statistical analysis was performed using non-parametric Student’s *t*-test to identify significance using GraphPad Prism5 software. ***p < 0.001, **p < 0.01, and *p < 0.05 were considered statistically significant.

**Figure 4:**
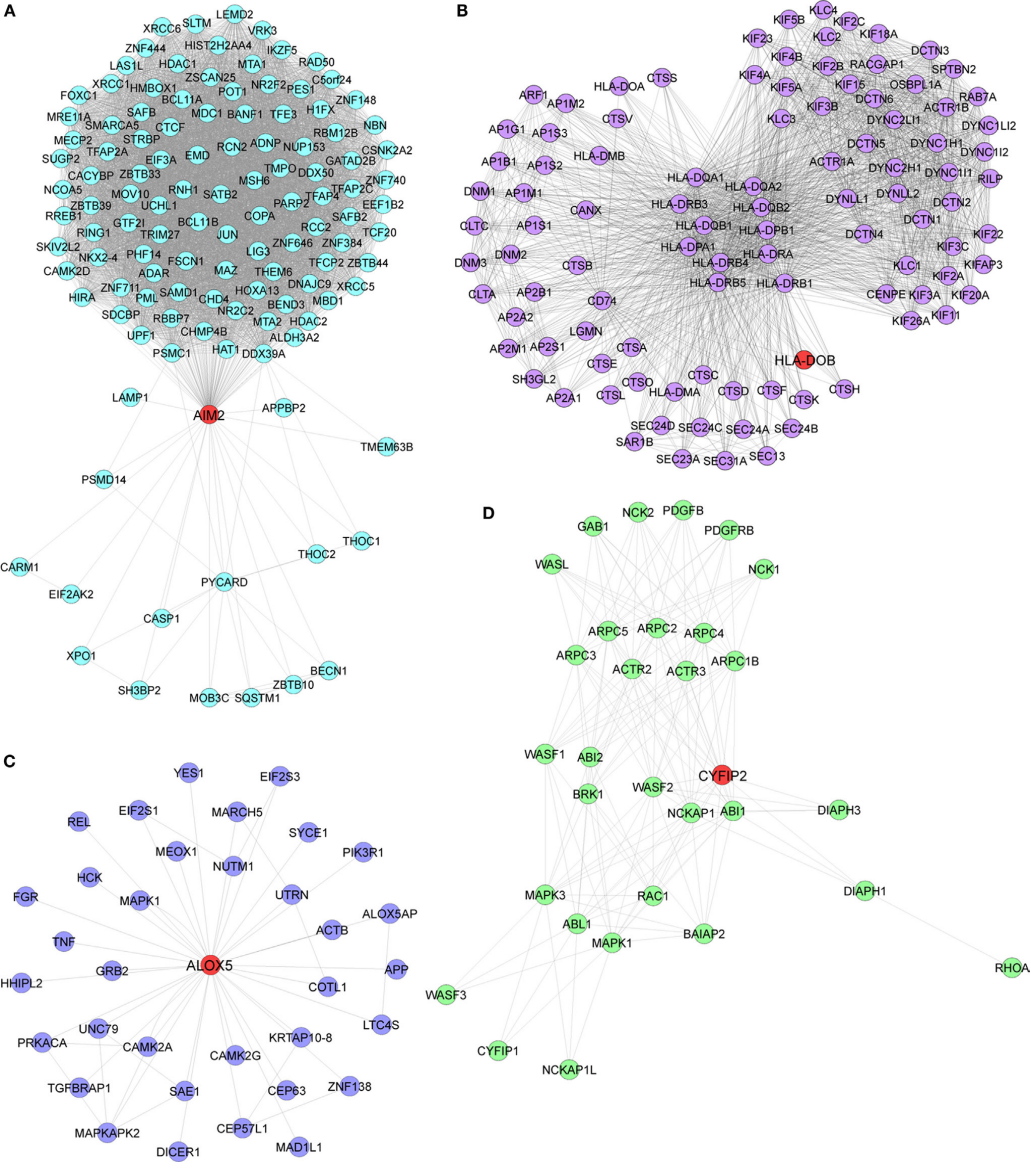
**Biological pathway networks of (A) AIM2, (B) HLA-DOB, (C) ALOX5, and (D) CYFIP2, generated using pathway commons and visualized on Cytoscape**.

**Figure 5:**
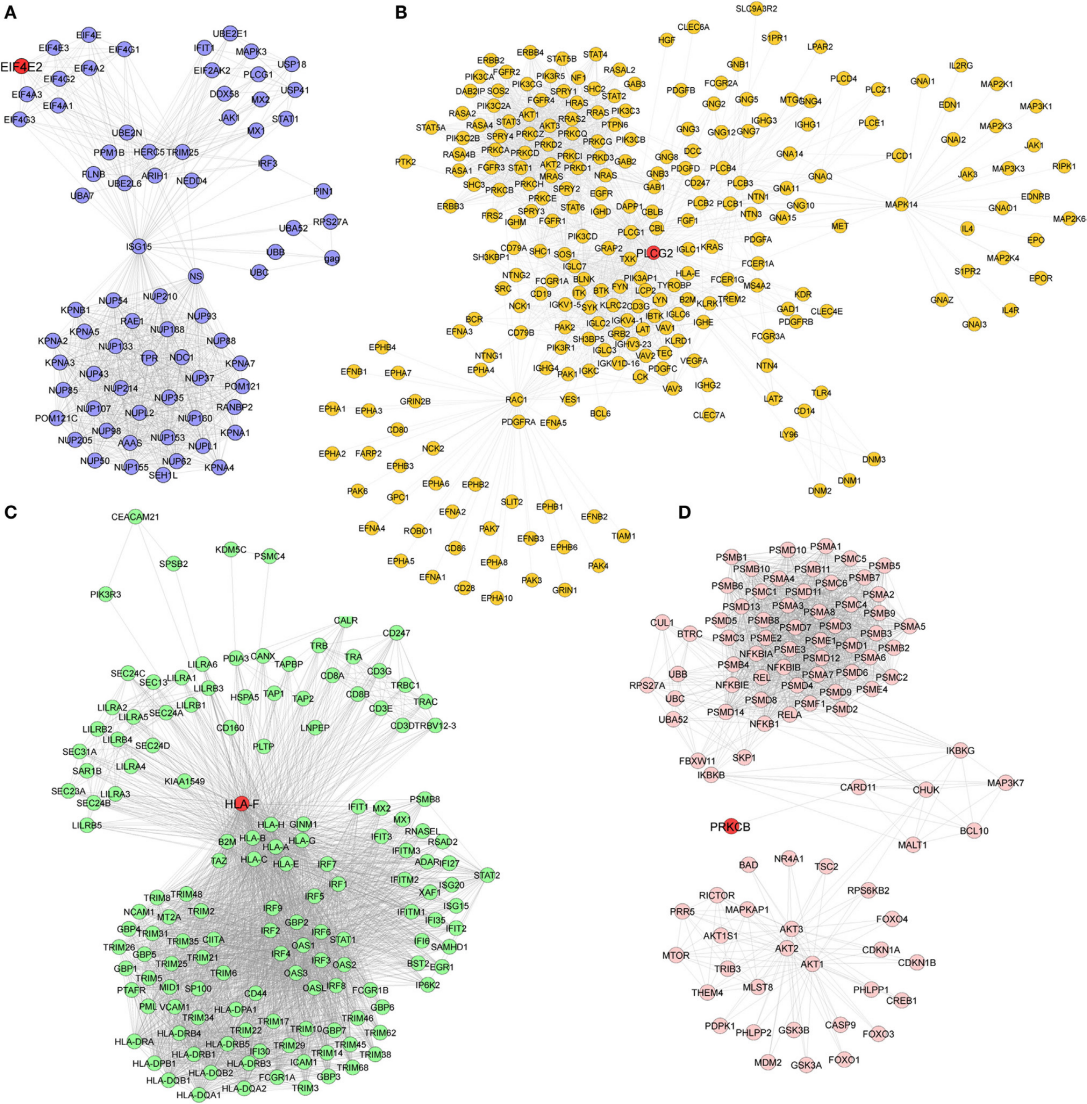
**Pathway networks of (A) EIF4E2, (B) PLCG2, (C) HLA-F, and (D) PRKCB**.

Pathway enrichment analysis of the meta-gene profile revealed a total of 20 genes involved in distinct pathways. Eight of the pathways associated genes (PLCG2, AIM2, ALOX-5, HLA-DOB, HLA-F, EIF4E2, PRKCB, and CYFIP2) were further studied by quantitative real-time PCR to validate the results obtained through the meta-analysis of publicly available RA patient’s microarray data sets. The genes for this in-depth study were selected by their diverse cellular functions, the magnitude of their differential expression, novelty (genes with unknown or less known functions), and for their potential relevance to disease. Quantitative RT-PCR for the genes quoted above was carried out using 19 patients clinically diagnosed with RA, and eight healthy volunteers were taken as HCs. Although the number of samples analyzed in the meta-analysis study and real-time PCR are limited, our results demonstrate that the relative expression levels analyzed through RT-qPCR for all the eight genes mentioned above were significantly upregulated in RA patient’s PBMCs as compared to HCs (Figures 3A–H). Of note, AIM2 (Figure 3B) and CYFIP2 (Figure 3H) were the two genes that were most significantly upregulated in PBMC samples of RA patients as compared to HCs. Overall, the RT-qPCR results were concordant with our meta-analysis study, suggesting the role of meta-analysis approach as a stupendous tool to identify differentially expressed gene signatures that might act as novel candidates to develop new therapeutic interventions against RA. Further, pathway networks of these validated candidate signatures were generated using Pathway Commons (31, 35–58) (www.pathwaycommons.org), a web resource for biological pathway data, and visualized using Cytoscape software (59) (Figures 4 and 5).

## Discussion

The etiology of RA like most of the immune-mediated disorders is multifactorial, which is influenced by genetic, environmental, hormonal, and immunological factors. However, genes, gene networks associated with RA onset and progression are not clearly defined. Our meta-analysis of RA gene expression data identified a robust meta-gene profile comprising 33 DEGs, which showed a consistent and significant up-or downregulation across all the data sets. To further understand the significance of these meta-genes in RA pathogenesis, we performed a GO functional and pathway enrichment analysis, which led to the identification of novel gene signatures that went unnoticed in previous RA studies. These genes include *PLCG2, HLA-DOB, HLA-F, EIF4E2*, and *CYFIP2*, which have been previously implicated in inflammation, antigen presentation, hypoxia, and apoptosis. Interestingly, expressions of these genes were also found to be significantly upregulated in PBMCs of clinical RA patients. It has been well established that the severity of RA is majorly dictated by the production of an array of pro-inflammatory cytokines in the synovial joint particularly cytokines like TNF and IL-1β, which triggers multiple cellular and immunological interactions as a result of excessive inflammation. Although biological therapies against TNF and other pro-inflammatory cytokines offer momentary clinical benefits, they are often associated with severe side effects (60). Therefore, it is decisive to identify and quantify combination of other genes, which might be helpful in controlling the massive inflammatory responses occurring during RA progression. Our meta-analysis study exemplifies the involvement of few inflammatory genes (PLCG2, AIM2, and ALOX-5), which might act as novel candidates for controlling inflammation during the disease condition (Figure 2B). Additionally, we have identified a previously unnoticed inflammatory gene, PLCG2 (phospholipase C-gamma 2), which has been found to be upregulated in all the microarray data sets as well in the clinical RA patients (Figures 2B and 3A). PLCG2, a transmembrane signaling enzyme, catalyzes the conversion of membrane phospholipid, phosphatidylinositol 4,5 bisphosphate into second messenger molecules inositol trisphosphate (IP3) and diacylglycerol (DAG) using calcium as the cofactor, thereby activating NLRP3 inflammasome. A gain of function mutation in PLCG2 gene is associated with phospholipase Cγ2 (PLCγ2)-associated antibody deficiency and immune dysregulation (APLAID), dominant inherited disorder, and auto-inflammation (61), thereby suggesting that PLCG2 might be a potential therapeutic target for controlling inflammation during RA pathogenesis. In consent with previous studies that have highlighted the role of AIM2 (absent in melanoma 2) and ALOX-5 (arachidonate 5-lipoxygenase) genes in RA pathogenesis, our study also demonstrated the enhanced expression of these genes in RA patients as compared to HCs (Figures 3B,C). AIM2 is known to induce the formation of caspase-1-activating inflammasome, thereby controlling the proteolytic maturation of pro-inflammatory cytokines IL-1β and IL-18 (62). Conditional deletion of AIM2 in RA mice model shows reduced inflammatory responses suggesting the role of AIM2 in controlling inflammation during RA (62). ALOX-5 is expressed basically in mature myeloid cells and developing/memory B-lymphocytes and is responsible for the generation of excessive leukotrienes (LTB4, LTC4, LTD4, and LTE4) in the synovial fluid of RA patients (63). Inhibitors of ALOX5 gene dampen TNF-α-induced NF-κβ, thereby abrogating synovial inflammation (63). ALOX5 in coordination with 5-LO-activating protein (FLAP) leads to the synthesis of LTA4, one of the critical intermediates of leukotrienes synthesis pathway (64). The long association of human leukocytic antigen (HLA) is confirmed in patients positive for rheumatoid factor. Appropriate antigen presentation or alteration in peptide affinity play a crucial role in promoting autoreactive adaptive immune responses (3). Intriguingly, our study for the first time has documented gene signatures HLA-DOB and HLA-F, which were found to be upregulated across all RA patient’s microarray data sets as well as in RA patients’ PBMCs (Figures 3D,E). HLA-DOB, a B-cell lineage, MHC-II-related molecule has been reported to have strong immunogenicity for human T-cells, one of its identified CTL epitopes HLA-DOB_232–240_ acts as a resilient immunotherapeutic candidate for targeting multiple myeloma (65). HLA-F, a non-classical MHC-I molecule, is currently enigmatic of all the HLA molecules; hence, its precise function remains elusive. Recent studies have demonstrated enhanced HLA-F expression in cancer stroma in the case of both breast and gastric cancers (66). HLA-F on B-cells was found to induce immune tolerance in tumor cells by interacting with inhibitory receptor ILT-2 and ILT-4 of the natural killer or CTLs, thereby blocking their cytotoxicity for the tumor cells (67). These findings categorically depict the immunological relevance of HLA gene signatures in multiple cancers; however, their abundance in RA patients and correlation with RA onset and progression needs to be completely deciphered.

Angiogenesis is one of the critical events in the perpetuation of RA (68). One of the important signals required for triggering angiogenesis during RA is hypoxia, which is created majorly as a result of synovial hyperplasia (10). Our meta-analysis study for the first time has identified EIF4E2 gene (eukaryotic initiation factor 4E2) encoding eIF4E2 protein, an mRNA cap binding homolog of eIF4E, which was found to be upregulated across all RA microarray data sets considered in our meta-analysis study as well as through RT-qPCR in RA patients (Figure 3F). eIF4E2 was found to be an inhibitor of translation in normal cells (69), while it binds with HIF-2α-RBM4 complex and drives the translation of proteins under hypoxic (low oxygen tension) conditions. Cancer cells exploit the eIF4E2-mediated protein synthesis to sustain hypoxic conditions and consequently grow to significant sizes (70). Perhaps, eIF4E2 might play a critical role in driving hypoxia-induced angiogenesis during RA, hence can act as a valuable pharmacological candidate for controlling RA. Further, our study also depicts the involvement of previously unnoticed genes such as protein kinase C-β, PRKCB (a negative regulator of BCR signaling) and cytoplasmic FMR1-interacting protein 2, CYFIP2 (inducer of p53 mediated apoptosis), which were also upregulated in all data sets and clinical RA patients (Figures 3G,H). CYFIP2 gene has a p53 responsive element that confers binding of p53 as well as the activation of some heterologous reporter. Inducible expression of CYFIP2 is responsible for caspase activation and cellular apoptosis (71). Although these novel immune signatures are abundantly expressed in RA patients, which categorically suggests the involvement of these gene/pathways in RA pathogenesis, still their functional relevance in terms of gene expression and disease outcome need to be deciphered. Nevertheless, identification of these novel gene signatures might provide a unique prospective to define novel molecular diagnostic candidates and ascertain potential pharmacological targets for the development of therapeutic interventions against RA.

## Author Contributions

All authors were involved in drafting the article or revising it critically for important intellectual content. All the authors read and approved the final version of the manuscript to be published. NK had full access to all of the data in the study and took responsibility for the integrity of the data and the accuracy of the data analysis. NK, SA, and JG participated in study conception and design. SA and JG were involved in the acquisition of data. SV and SN were responsible for recruitment and clinical evaluation of RA patients. NK, SA, and JG participated in analysis and interpretation of data and manuscript writing.

## Conflict of Interest Statement

The authors declare that the research was conducted in the absence of any commercial or financial relationships that could be construed as a potential conflict of interest.
