# Supplementary material for: A Comprehensive Gene Expression Meta-analysis Identifies Novel Immune Signatures in Rheumatoid Arthritis Patients
Source: Front Immunol. 2017 Feb 2;8:74. doi: 10.3389/fimmu.2017.00074 (PMC5288395; doi:10.3389/fimmu.2017.00074)
Supplement: Supplementary file 1 [file Presentation_1.PDF]

Supplementary Material

A comprehensive gene expression meta-analysis identifies novel immune signatures in Rheumatoid Arthritis patients

Sumbul Afroz [1+], Jeevan Giddaluru[1+], Sandeep Vishwakarma [2], Saima Naz [2], Aleem Ahmed Khan[2], and Nooruddin Khan[1*]

*Correspondence:
Nooruddin Khan
(noor@uohyd.ac.in)

+Sumbul Afroz and Jeevan Giddaluru contributed equally to this work.

1. SUPPLEMENTARY FIGURES AND TABLES

1.1 Figures

Figure S1. Reverse expression of genes between PBMC and Synovial tissue data sets.

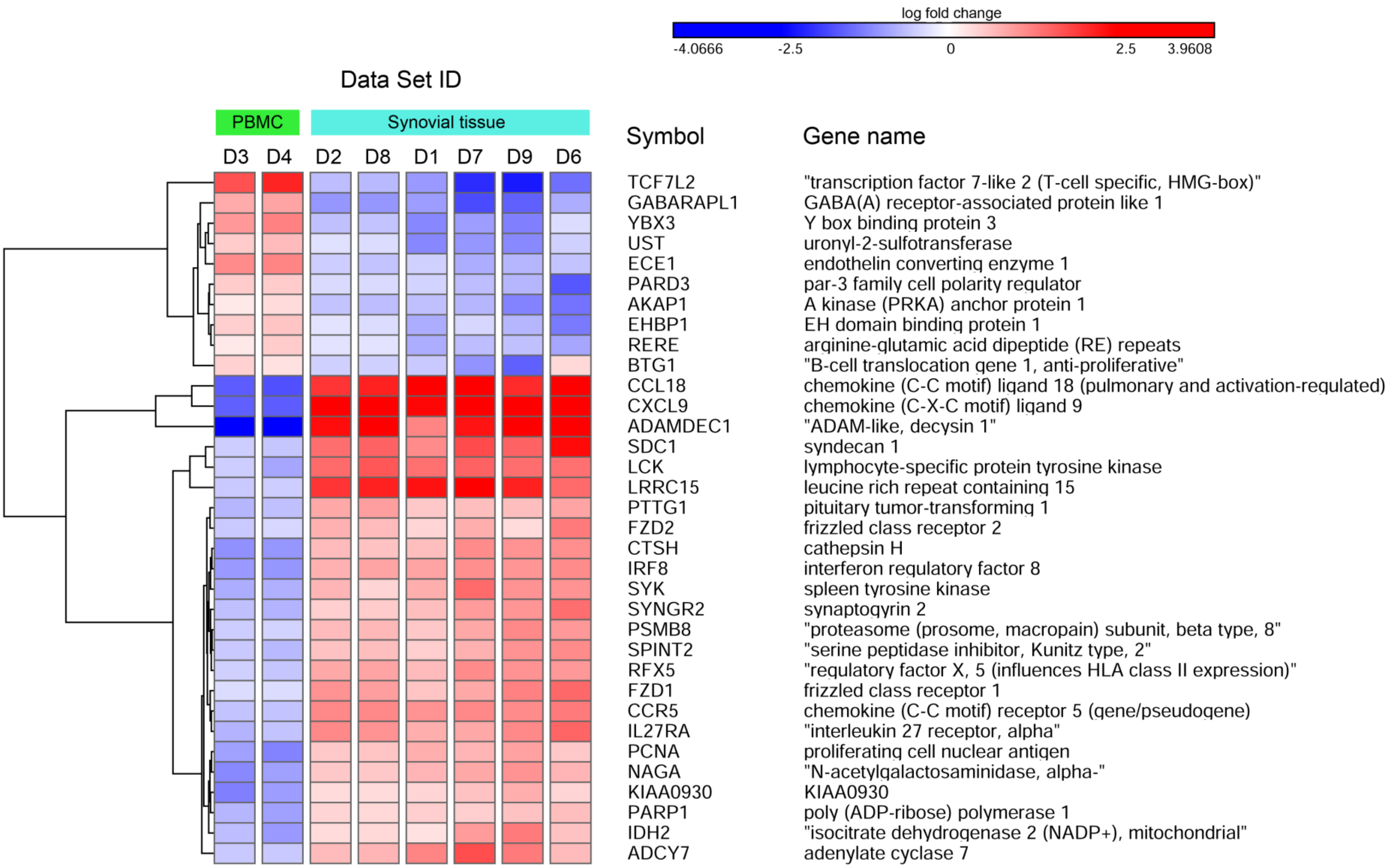

Figure S2. Heat map representing additional meta-genes obtained after excluding data set 1 [Consistently expressed (n=80) and reversely expressed (n=70) b/w PBMCs and Synovial data sets]

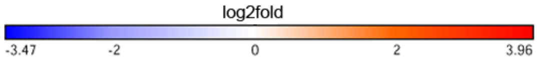

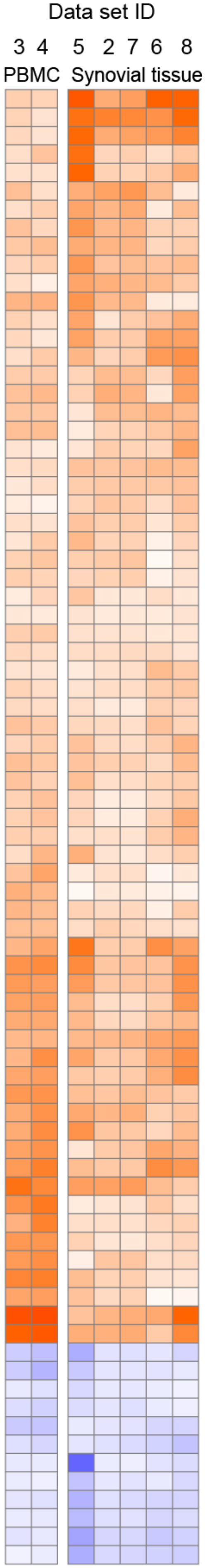

Symbol	Gene name
"FKBP11"	"FK506 binding protein 11, 19 kDa"
"TPD52"	"tumor protein D52"
"SEMA4D"	"sema domain, immunoglobulin domain (Ig), transmembrane domain (TM) and short cytoplasmic domain, (semaphorin)
"APOBEC3B"	"apolipoprotein B mRNA editing enzyme, catalytic polypeptide-like 3B"
"WIPF1"	"WAS/WASL interacting protein family, member 1"
"KIAA0125"	"KIAA0125"
"ASPM"	"asp (abnormal spindle) homolog, microcephaly associated (Drosophila)"
"CD72"	"CD72 molecule"
"TOX"	"thymocyte selection-associated high mobility group box"
"OSBPL3"	"oxysterol binding protein-like 3"
"TNFAIP8"	"tumor necrosis factor, alpha-induced protein 8"
"TRAT1"	"T cell receptor associated transmembrane adaptor 1"
"FLI1"	"Fli-1 proto-oncogene, ETS transcription factor"
"TRIM22"	"tripartite motif containing 22"
"ATXN1"	"ataxin 1"
"AGA"	"aspartylglucosaminidase"
"JAK2"	"Janus kinase 2"
"DENND1B"	"DENN/MADD domain containing 1B"
"AREL1"	"apoptosis resistant E3 ubiquitin protein ligase 1"
"PEX2"	"peroxisomal biogenesis factor 2"
"TAPBPL"	"TAP binding protein-like"
"PLCL2"	"phospholipase C-like 2"
"RACGAP1"	"Rac GTPase activating protein 1"
"ACSL5"	"acyl-CoA synthetase long-chain family member 5"
"BUB1B"	"BUB1 mitotic checkpoint serine/threonine kinase B"
"DTL"	"denticleless E3 ubiquitin protein ligase homolog (Drosophila)"
"SYT11"	"synaptotagmin XI"
"RNF44"	"ring finger protein 44"
"PDE6D"	"phosphodiesterase 6D, cGMP-specific, rod, delta"
"TRIM21"	"tripartite motif containing 21"
"SPG11"	"spastic paraplegia 11 (autosomal recessive)"
"MSL3"	"male-specific lethal 3 homolog (Drosophila)"
"VRK2"	"vaccinia related kinase 2"
"SPCS1"	"signal peptidase complex subunit 1 homolog (S. cerevisiae)"
"STK38"	"serine/threonine kinase 38"
"PSEN1"	"presenilin 1"
"DDX58"	"DEAD (Asp-Glu-Ala-Asp) box polypeptide 58"
"IRF9"	"interferon regulatory factor 9"
"UBE2L3"	"ubiquitin-conjugating enzyme E2L 3"
"TM9SF2"	"transmembrane 9 superfamily member 2"
"TXNDC9"	"thioredoxin domain containing 9"
"MINPP1"	"multiple inositol-polyphosphate phosphatase 1"
"SRD5A1"	"steroid-5-alpha-reductase, alpha polypeptide 1 (3-oxo-5 alpha-steroid delta 4-dehydrogenase alpha 1)"
"ZNF107"	"zinc finger protein 107"
"IQCG"	"IQ motif containing G"
"TPK1"	"thiamin pyrophosphokinase 1"
"EVI2B"	"ecotropic viral integration site 2B"
"ENTPD1"	"ectonucleoside triphosphate diphosphohydrolase 1"
"UBE2J1"	"ubiquitin-conjugating enzyme E2, J1"
"CD46"	"CD46 molecule, complement regulatory protein"
"RAB31"	"RAB31, member RAS oncogene family"
"CASP1"	"caspase 1, apoptosis-related cysteine peptidase"
"TAPBP"	"TAP binding protein (tapasin)"
"SEPT6"	"septin 6"
"LRMP"	"lymphoid-restricted membrane protein"
"IL15"	"interleukin 15"
"TLR8"	"toll-like receptor 8"
"MICAL2"	"microtubule associated monooxygenase, calponin and LIM domain containing 2"
"SMCO4"	"single-pass membrane protein with coiled-coil domains 4"
"CD38"	"CD38 molecule"
"DNAJC15"	"DnaJ (Hsp40) homolog, subfamily C, member 15"
"UBE2A"	"ubiquitin-conjugating enzyme E2A"
"TMEM50A"	"transmembrane protein 50A"
"MDM1"	"Mdm1 nuclear protein homolog (mouse)"
"SIRPG"	"signal-regulatory protein gamma"
"IGF2BP3"	"insulin-like growth factor 2 mRNA binding protein 3"
"TNFSF10"	"tumor necrosis factor (ligand) superfamily, member 10"
"GUCY1B3"	"guanylate cyclase 1, soluble, beta 3"
"ESF1"	"ESF1, nucleolar pre-rRNA processing protein, homolog (S. cerevisiae)"
"TAF15"	"TAF15 RNA polymerase II, TATA box binding protein (TBP)-associated factor, 68kDa"
"TIMM44"	"translocase of inner mitochondrial membrane 44 homolog (yeast)"
"SNTA1"	"syntrophin, alpha 1"
"PXDC1"	"PX domain containing 1"
"PITX1"	"paired-like homeodomain 1"
"KLHL24"	"kelch-like family member 24"
"HIF3A"	"hypoxia inducible factor 3, alpha subunit"
"DDX24"	"DEAD (Asp-Glu-Ala-Asp) box helicase 24"
"ID4"	"inhibitor of DNA binding 4, dominant negative helix-loop-helix protein"
"FOXC1"	"forkhead box C1"
"SON"	"SON DNA binding protein"

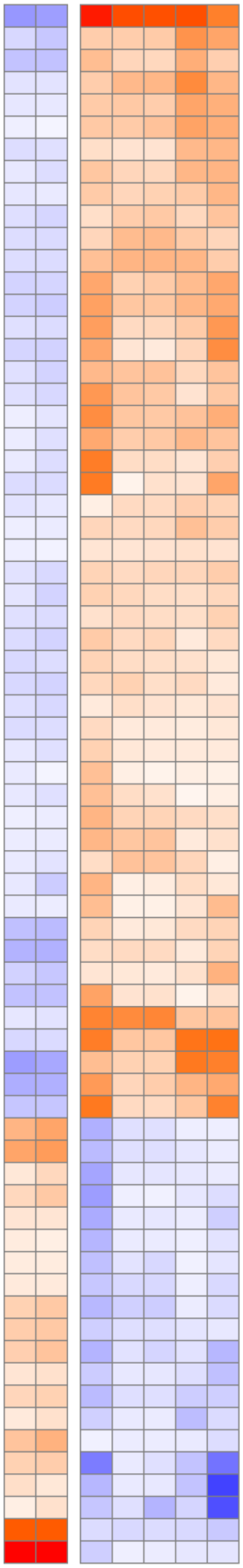

"SLAMF8"	"SLAM family member 8"
"CRLF3"	"cytokine receptor-like factor 3"
"ACP2"	"acid phosphatase 2, lysosomal"
"GNPTAB"	"N-acetylglucosamine-1-phosphate transferase, alpha and beta subunits"
"CREBL2"	"cAMP responsive element binding protein-like 2"
"YME1L1"	"YME1-like 1 ATPase"
"LASP1"	"LIM and SH3 protein 1"
"PAPSS1"	"3'-phosphoadenosine 5'-phosphosulfate synthase 1"
"ATOX1"	"antioxidant 1 copper chaperone"
"RFTN1"	"raftlin, lipid raft linker 1"
"MCM5"	"minichromosome maintenance complex component 5"
"PLEKHO1"	"pleckstrin homology domain containing, family O member 1"
"OPN3"	"opsin 3"
"LEPROTL1"	"leptin receptor overlapping transcript-like 1"
"SEL1L"	"sel-1 suppressor of lin-12-like (C. elegans)"
"TRAM1"	"translocation associated membrane protein 1"
"GGH"	"gamma-glutamyl hydrolase (conjugase, folylpolygammaglutamyl hydrolase)"
"ETNK1"	"ethanolamine kinase 1"
"ERAP1"	"endoplasmic reticulum aminopeptidase 1"
"TRPV2"	"transient receptor potential cation channel, subfamily V, member 2"
"MGAT4A"	"mannosyl (alpha-1,3-)-glycoprotein beta-1,4-N-acetylglucosaminyltransferase, isozyme
"MOB1A"	"MOB kinase activator 1A"
"HPS5"	"Hermansky-Pudlak syndrome 5"
"ARHGEF18"	"Rho/Rac guanine nucleotide exchange factor (GEF) 18"
"KIAA0226"	"KIAA0226"
"CRELD2"	"cysteine-rich with EGF-like domains 2"
"HLA-E"	"major histocompatibility complex, class I, E"
"UBAC1"	"UBA domain containing 1"
"MCM2"	"minichromosome maintenance complex component 2"
"APEH"	"acylaminoacyl-peptide hydrolase"
"PDCD1LG2"	"programmed cell death 1 ligand 2"
"KHNYN"	"KH and NYN domain containing"
"RITA1"	"RBPJ interacting and tubulin associated 1"
"SNX11"	"sorting nexin 11"
"DCAF7"	"DDB1 and CUL4 associated factor 7"
"ZNF557"	"zinc finger protein 557"
"SLC12A8"	"solute carrier family 12, member 8"
"WNT5A"	"wingless-type MMTV integration site family, member 5A"
"RABEP2"	"rabaptin, RAB GTPase binding effector protein 2"
"HLA-A"	"major histocompatibility complex, class I, A"
"TRIM27"	"tripartite motif containing 27"
"C19orf10"	"chromosome 19 open reading frame 10"
"RAD17"	"RAD17 homolog (S. pombe)"
"RER1"	"retention in endoplasmic reticulum sorting receptor 1"
"RAD51AP1"	"RAD51 associated protein 1"
"IL21R"	"interleukin 21 receptor"
"CECR1"	"cat eye syndrome chromosome region, candidate 1"
"CSF1R"	"colony stimulating factor 1 receptor"
"SGPL1"	"sphingosine-1-phosphate lyase 1"
"SSR1"	"signal sequence receptor, alpha"
"MAP7D3"	"MAP7 domain containing 3"
"HIP1"	"huntingtin interacting protein 1"
"MAP4"	"microtubule-associated protein 4"
"NFIB"	"nuclear factor I/B"
"GPATCH8"	"G patch domain containing 8"
"WDR60"	"WD repeat domain 60"
"AKAP13"	"A kinase (PRKA) anchor protein 13"
"SFSWAP"	"splicing factor, suppressor of white-apricot homolog (Drosophila)"
"TOP1"	"topoisomerase (DNA) I"
"TOX4"	"TOX high mobility group box family member 4"
"PRRC2C"	"proline-rich coiled-coil 2C"
"TRAK2"	"trafficking protein, kinesin binding 2"
"SMAD3"	"SMAD family member 3"
"EIF1"	"eukaryotic translation initiation factor 1"
"BAZ2A"	"bromodomain adjacent to zinc finger domain, 2A"
"PIK3R1"	"phosphoinositide-3-kinase, regulatory subunit 1 (alpha)"
"SLC16A7"	"solute carrier family 16 (monocarboxylate transporter), member 7"
"SRRM2"	"serine/arginine repetitive matrix 2"
"SLC25A37"	"solute carrier family 25 (mitochondrial iron transporter), member 37"
"LGALSL"	"lectin, galactoside-binding-like"

1.2 Tables

Table S1. GO Biological processes

Term	Combined Score	Genes
regulation of B cell receptor signaling pathway (GO:0050855)	15.41722538	PTPRC;PRKCB;LPXN
B cell receptor signaling pathway (GO:0050853)	10.02939828	PTPRC;PRKCB;PLCG2
regulation of antigen receptor-mediated signaling pathway (GO:0050854)	10.12064039	PTPRC;PRKCB;LPXN
cellular calcium ion homeostasis (GO:0006874)	9.119069293	CD52;PTPRC;EDNRB;PRKCB;PLCG2
cellular divalent inorganic cation homeostasis (GO:0072503)	9.078084541	CD52;PTPRC;EDNRB;PRKCB;PLCG2
calcium ion homeostasis (GO:0055074)	9.111679637	CD52;PTPRC;EDNRB;PRKCB;PLCG2
cellular chemical homeostasis (GO:0055082)	9.307192349	CD52;PTPRC;EDNRB;PRKCB;UCP2;PLCG2
positive regulation of cytosolic calcium ion concentration (GO:0007204)	8.772884667	CD52;PTPRC;EDNRB;PLCG2
divalent inorganic cation homeostasis (GO:0072507)	9.051165821	CD52;PTPRC;EDNRB;PRKCB;PLCG2
positive regulation of antigen receptor-mediated signaling pathway (GO:0050857)	8.569901132	PTPRC;PRKCB
cytosolic calcium ion homeostasis (GO:0051480)	8.140754016	CD52;PTPRC;EDNRB;PLCG2
mast cell activation (GO:0045576)	8.086382903	RHOH;RASGRP1
cellular metal ion homeostasis (GO:0006875)	7.851137685	CD52;PTPRC;EDNRB;PRKCB;PLCG2
negative regulation of homeostatic process (GO:0032845)	6.966597817	PTPRC;UCP2;PLCG2
leukocyte activation (GO:0045321)	7.512739974	PTPRC;PRKCB;PLCG2;RHOH;RASGRP1
cellular cation homeostasis (GO:0030003)	7.581739575	CD52;PTPRC;EDNRB;PRKCB;PLCG2
positive regulation of T cell mediated cytotoxicity (GO:0001916)	8.41492598	PTPRC;HLA-F
cytolysis (GO:0019835)	7.99537505	GZMA;GZMH
cellular ion homeostasis (GO:0006873)	7.528936676	CD52;PTPRC;EDNRB;PRKCB;PLCG2
regulation of T cell mediated cytotoxicity (GO:0001914)	7.838598341	PTPRC;HLA-F
metal ion homeostasis (GO:0055065)	7.143243636	CD52;PTPRC;EDNRB;PRKCB;PLCG2
immune response-regulating cell surface receptor signaling pathway (GO:0002768)	10.04878821	CYFIP2;PTPRC;PRKCB;PLCG2;RASGRP1
regulation of protein oligomerization (GO:0032459)	7.490271004	AIM2;PEX5
release of sequestered calcium ion into cytosol (GO:0051209)	7.137698214	PTPRC;PLCG2
negative regulation of sequestering of calcium ion (GO:0051283)	7.128076376	PTPRC;PLCG2
cation homeostasis (GO:0055080)	6.70236087	CD52;PTPRC;EDNRB;PRKCB;PLCG2
regulation of sequestering of calcium ion (GO:0051282)	7.010675692	PTPRC;PLCG2
antigen receptor-mediated signaling pathway (GO:0050851)	6.225549166	PTPRC;PRKCB;PLCG2
B cell activation (GO:0042113)	5.985923215	PTPRC;PRKCB;PLCG2

Table S2. GO Molecular functions

Term	Combined Score	Genes
antigen binding (GO:0003823)	6.729254	HLA-F;HLA-DOB
MHC class II receptor activity (GO:0032395)	4.379259	HLA-DOB
GTPase inhibitor activity (GO:0005095)	4.270184	RHOH
RNA cap binding (GO:0000339)	4.076628	EIF4E2
peptide binding (GO:0042277)	3.977479	EDNRB;PEX5;HLA-F
phosphatidate phosphatase activity (GO:0008195)	3.975554	PPAP2B
amide binding (GO:0033218)	3.968773	EDNRB;PEX5;HLA-F
histone kinase activity (GO:0035173)	3.965963	PRKCB
MHC class II protein complex binding (GO:0023026)	3.873877	HLA-DOB
phosphoric ester hydrolase activity (GO:0042578)	3.857543	PTPRC;PPAP2B;PLCG2
protein kinase C activity (GO:0004697)	3.835392	PRKCB
angiotensin receptor binding (GO:0031701)	3.812321	EDNRB
MHC protein complex binding (GO:0023023)	3.776218	HLA-DOB
translation factor activity, nucleic acid binding (GO:0008135)	3.767175	EEF1D;EIF4E2
transmembrane receptor protein tyrosine phosphatase activity (GO:0005001)	3.760755	PTPRC
type 1 angiotensin receptor binding (GO:0031702)	3.738759	EDNRB
transmembrane receptor protein phosphatase activity (GO:0019198)	3.670221	PTPRC
heparan sulfate proteoglycan binding (GO:0043395)	3.60922	PTPRC
serine-type endopeptidase activity (GO:0004252)	3.516884	GZMA;GZMH

Table S3. Top pathways enriched

**Reactome Pathway**	**P-value**	**Hit Genes**
Immune System	0.0001	RASGRP1,IL2RG,HLA-DOB,PTPRC,AIM2,HLA-F,PRKCB, PSME2,PLCG2,CYFIP2,EIF4E2
Adaptive Immune System	0.0011	RASGRP1,HLA-DOB,PTPRC,HLA-F,PRKCB,PSME2,PLCG2
Platelet activation, signaling and aggregation	0.0025	RASGRP1,IL2RG,PRKCB,PLCG2
GPVI-mediated activation cascade	0.0076	IL2RG,PLCG2
Hemostasis	0.0088	RASGRP1,IL2RG,MAFF,PRKCB,PLCG2
The AIM2 inflammasome	0.009	AIM2
Downstream signaling events of B Cell Receptor (BCR)	0.0123	RASGRP1,PRKCB,PSME2
Signaling by the B Cell Receptor (BCR)	0.0138	RASGRP1,PRKCB,PSME2,PLCG2
Synthesis of Lipoxins (LX)	0.0149	ALOX5
Synthesis of 5-eicosatetraenoic acids	0.0149	ALOX5
Activation of RAS in B cells	0.0149	RASGRP1
Disinhibition of SNARE formation	0.0149	PRKCB
ER-Phagosome pathway	0.0156	HLA-F,PSME2
Semaphorin interactions	0.016	PTPRC,MYH11
Activation of NF-kappaB in B cells	0.0165	PRKCB,PSME2
Interleukin-7 signaling	0.0178	IL2RG
Antigen processing-Cross presentation	0.021	HLA-F,PSME2
Depolymerisation of the Nuclear Lamina	0.0266	PRKCB
Endosomal/Vacuolar pathway	0.0266	HLA-F
Class I MHC mediated antigen processing & presentation	0.0294	HLA-F,PSME2
VEGFA-VEGFR2 Pathway	0.0324	PRKCB,CYFIP2
Synthesis of Leukotrienes (LT) and Eoxins (EX)	0.0325	ALOX5
Caspase-mediated cleavage of cytoskeletal proteins	0.0354	GSN
Signaling by VEGF	0.0376	PRKCB,CYFIP2
Trafficking of GluR2-containing AMPA receptors	0.0469	PRKCB
Rap1 signaling	0.0469	RASGRP1
Cytokine signaling in Immune system	0.0483	IL2RG,HLA-F,EIF4E2
Inflammasomes	0.0498	AIM2
Other semaphorin interactions	0.0554	PTPRC

Table S4. Gene-specific primers

S no.	Genes	Primers
1.	PLCG2	FP 5’ CATCCTATATGGCACTCAGTTCG 3’<br>RP 5’ TCCTGGTGTAAGATTTTCAAGCC 3’
2.	ALM2	FP 5’ AGCAAGATATTATCGGCACAGTG 3’<br>RP 5’ GTTCAGCGGGACATTAACCTT 3’
3.	ALOX5	FP 5’ CTCAAGCAACACCGACGTAAA 3’<br>RP 5’ CCTTGTGGCATTTGGCATCG 3’
4.	HLADOB	FP 5’ ATCTGACCCGACTGGATTCCT 3’<br>RP 5’ GCACCTTTTCTGTCCCGTTG 3’
5.	HLA-F	FP 5’ TGGCCCTGACCGATACTTG 3’<br>RP 5’ GCAGGAATTGCGTGTCGTC 3’
6.	EIF4E2	FP 5’ ACAACAAGTTCGACGCTTTGA 3’<br>RP 5’ TCTCTTGCTACTGCTCTGATTCT 3’
7.	PRKCB	FP 5’ AGCCCCACGTTTTGTGACC 3’<br>RP 5’ GCTGGGAACATTCATCACGC 3’
8.	CYFIP2	FP 5’ CAAGGTACATTGAGCAGGCTAC 3’<br>RP 5’ CTCGTTGCATTTCACCTGGG 3’
